# A *cis*-eQTL of *HLA-DRB1* and a frameshift mutation of *MICA* contribute to the pattern of association of HLA alleles with cervical cancer

**DOI:** 10.1002/cam4.192

**Published:** 2014-02-12

**Authors:** Dan Chen, Ulf Gyllensten

**Affiliations:** Department of Immunology, Genetics and Pathology, Rudbeck Laboratory, Science for Life Laboratory Uppsala, Uppsala UniversityUppsala, Sweden

**Keywords:** Cervical cancer, *cis*-eQTL, frameshift mutation, HLA, MICA

## Abstract

The association of classic human leukocyte antigen (HLA) alleles with risk of cervical cancer has been extensively studied, and a protective effect has consistently been found for DRB1*1301, DQA1*0103, and/or DQB1*0603 (these three alleles are in perfect linkage disequilibrium [LD] and often occur on the same haplotype in Europeans), while reports have differed widely with respect to the effect of HLA-B*07, DRB1*1501, and/or DQB1*0602 (the last two alleles are also in perfect LD in Europeans). It is not clear whether the reported HLA alleles are responsible for the differences in cervical cancer susceptibility, or if functional variants at other locations within the major histocompatibility complex (MHC) region may explain the effect. In order to assess the relative contribution of both classic HLA alleles and single-nucleotide polymorphisms (SNPs) within the MHC region to cervical cancer susceptibility, we have imputed classic HLA alleles in 1034 cervical cancer patients and 3948 controls in a Swedish population for an integrated analysis. We found that the protective haplotype DRB1*1301-DQA1*0103-DQB1*0603 has a direct effect on cervical cancer and always occurs together with the C allele of a *HLA-DRB1 cis*-eQTL (rs9272143), which increases the expression of *HLA-DRB1*. The haplotype rs9272143*C*-DRB1*1301-DQA1*0103-DQB1*0603 conferred the strongest protection against cervical cancer (odds ratio [OR] = 0.41, 95% confidence interval [CI] = 0.32–0.52, *P *= 6.2 × 10^−13^). On the other hand, the associations with *HLA*-B*0702 and DRB1*1501-DQB1*0602 are attributable to the joint effects of both the *HLA-DRB1 cis*-eQTL (rs9272143) and a frameshift mutation (G inserion of rs67841474, also known as A5.1) of the MHC class I polypeptide-related sequence A gene (*MICA*). Variation in LD between the classic HLA loci, rs9272143 and rs67841474 between populations may explain the different associations of *HLA*-B*07 and DRB1*1501-DQB1*0602 with cervical cancer between studies. The mechanism suggested may also explain similar inconsistent results for other HLA-associated diseases.

## Introduction

Worldwide, cervical cancer is the third most common cancer among women 1. Persistent infection with high-risk human papillomavirus (HPV) is the main causal factor of cervical cancer and its precursor lesions, cervical intraepithelial neoplasia (CIN), where CIN III is considered the same as carcinoma in situ (CIS) or Stage 0 cervical cancer 2. The genetic variability in the host plays an important role in the persistence of HPV infections and progression to cervical cancer, especially genetic factors that control the immune response 3. Human leukocyte antigen (HLA) molecules are responsible for the presentation of foreign antigens to the immune system, and play a central role in the immune recognition and subsequent clearance of virally infected cells 4. Classic HLA alleles, defined by genetic variation within the peptide-binding site of HLA genes, are believed to influence the ability to bind and present different peptide antigens 5,6. Numerous studies have studied the association of specific HLA alleles with risk of (pre) neoplastic cervical disease, but with variable results between studies. Some studies have found the HLA class II DRB1*1501 and/or DQB1*0602 (two alleles that are in perfect linkage disequilibrium [LD] and often occur on the same haplotype in Europeans) 5,6 as well as class I HLA-B*07 7–9 to increase risk of cervical disease, while other studies have failed to confirm these associations 10–12 and some have even found inverse associations 8,13,14. In contrast, studies have consistently reported DRB1*1301, DQA1*0103, and/or DQB1*0603 (these three alleles are also in perfect LD in Europeans) to reduce risk of cervical disease 5,6. Most of these studies have not taken into account the complex LD pattern that extends across multiple HLA and non-HLA genes within the major histocompatibility complex (MHC) region 15, and the need to control rigorously for population stratification. It is, therefore, not clear whether the reported HLA alleles are responsible for the differences in cervical cancer susceptibility, or if functional variants at other locations may explain the effect.

It has recently been shown that single-nucleotide polymorphism (SNP) data within the 6p21 region can be used to impute alleles at key classic HLA class I (*HLA-A*, *HLA-B,* and *HLA-C*) and class II (*HLA-DQA1*, *HLA-DQB1,* and *HLA-DRB1*) loci with accuracy that exceeds 90% at the four-digit level 16. In order to assess the relative contribution of both classic HLA alleles and SNP variation to cervical cancer susceptibility, we used existing SNP data from a previous genome-wide association study (GWAS) of cervical cancer 17 to impute classic HLA alleles in 1034 cervical cancer patients and 3948 controls for an integrated analysis.

## Materials and Methods

### Study population and genotyping

Subjects included in this study were from a previous GWAS of cervical cancer in a Swedish population, and were recruited from two studies, the CervixCan I study and the TwinGene study 17. In the CervixCan study, cervical cancer patients were selected from families with at least two affected women from Sweden who were born after 1940 and reported to the Swedish Cancer Registry before 1993. Details on study designs and subject recruitment have been described previously 18. The CervixCan study was further divided into two parts, that is, the CervixCan I study that comprises cases who are the sole participants of their family and the CervixCan II study that comprises individuals with more than one first-degree relative also participating. 766 sole participants (720 CIS and 46 invasive carcinoma) from the CervixCan I study were included in this study. Direct genotyping of *HLA-B*, *-DQB1,* and *-DRB1* was performed in 576 cervical cancer patients from the CervixCan II study 18. The TwinGene study is a population-based Swedish study of twins born between 1911 and 1958. In total, 9896 subjects were genotyped consecutively with those from the CervixCan I study using Illumina HumanOmniExpress BeadChip (731,422, SNPs). Among these subjects, 309 unrelated cervical cancer cases (288 CIS and 21 invasive carcinoma) were further included in this study. One female singleton was then randomly selected from each twin pair without cervical cancer, resulting in 4014 unrelated cervical cancer-free females who were included as controls. The details of quality control (QC) have been described previously 17. Briefly, after stringent QC, data from 1034 cervical cancer patients (971 CIS and 63 invasive carcinoma) and 3948 control subjects were available for 632,668 SNPs with an overall call rate of 99.92%. Utilizing a set of 17,386 SNPs evenly distributed across the genome and in low LD (pairwise *r*^2^ < 0.02) that passed QC, principal components analysis (PCA) was performed using EIGENSTRAT package (Broad Institute of Harvard and MIT, New York, USA) 19 to identify population stratification. Nine significant eigenvectors were identified based on the Tracy–Widom statistic (*P* < 0.05) from the PCA.

### Statistical analysis for SNPs

For single-SNP analysis, we considered the extended MHC to be defined by a 7.64 Mb region bordered by the histone cluster 1, H2aa gene (*HIST1H2AA*) and ribosomal protein L12 pseudogene 1 (*RPL12P1*) 20 (rs4711095 at 25,726,774 bp and rs1547668 at 33,775,446 bp, respectively) at the telomeric and centromeric ends of 6p21, respectively, which include 5976 genotyped SNPs in this study. All positions are with respect to the GRCh37.p10/hg19 assembly of the Human Genome. The association between each SNP and risk of cervical cancer was estimated by the odds ratio (OR) per minor allele and 95% confidence interval (CI) using multivariate unconditional logistic regression in allelic test with adjustment for population stratification by including nine informative eigenvectors as covariates, generated by PCA.

### Imputation of classic HLA alleles and statistical analysis

From a reference database of SNP haplotypes carrying known HLA alleles, we imputed HLA genotypes for all the subjects using HLA*IMP 16. The reference database combines classic HLA data from the HapMap Project and the 1958 Birth Cohort 16. Imputation was performed for three class I (*HLA-A* [*n* = 2474 reference samples], *-B* [*n* = 3 090], *-C* [*n* = 2022]), and three class II (*DQA1* [*n* = 175], *-DQB1* [*n* = 2629], and *-DRB1* [*n* = 2665]) loci. Imputation accuracy was assessed through a cross-validation analysis of the training data. Training was performed on two-thirds of the reference panel with known HLA types and tested on the remaining third at the SNPs chosen for imputation in this study. Thresholding calls at a posterior probability of 0.7 provided call rates of between 0.91 (*HLA-DRB1*) and 0.99 (*HLA-DQB1*) and accuracy of ≥0.95 for all loci, suggesting that the imputed HLA data are reliable. To ensure the accuracy of prediction, we restricted our case–control analysis to those alleles that were predicted with a posterior probability more than 90%. The association between each HLA allele/haplotype and cervical cancer was then estimated by ORs and 95% CIs using unconditional logistic regression in allelic test. Incorporating these probabilities directly into the general logistic regression framework resulted in similar association results (data not shown). Adjustment for population stratification was performed by including nine informative eigenvectors as covariates in the logistic regression, generated by PCA. Statistical analyses were all performed using SAS 9.3 software (SAS Institute, Cary, NC), with two-sided tests. Pairwise LD was estimated by Haploview 21.

## Results and Discussion

After imputation of classic HLA loci, 27 *HLA-A* alleles, 48 *HLA-B* alleles, 21 *HLA-C* alleles, seven *HLA-DQA1* alleles, 18 *HLA-DQB1* alleles, and 34 *HLA-DRB1* alleles were observed in this study. Association analysis was performed for 5976 SNPs and 155 classic HLA alleles within the extended MHC region, resulting in a significance threshold of *P* = 8.1 × 10^−6^ based on Bonferroni correction.

A total of 161 SNPs were associated with risk of cervical cancer at *P* < 8.1 × 10^−6^. The strongest signal of association was attained for rs9272143 between *HLA-DRB1* and *HLA-DQA1* (OR = 0.65, 95% CI = 0.59–0.72, *P* = 2.8 × 10^−17^). When conditioning upon rs9272143, residual association was detected at some SNPs in this region, with the most significant signal occurring at rs2516448 adjacent to the MHC class I polypeptide-related sequence A gene (*MICA*) (OR = 1.46, 95% CI = 1.32–1.61, *P* = 8.6 × 10^−14^ compared with OR = 1.44, 95% CI = 1.30–1.58, *P* = 5.8 × 10^−13^ in the unconditional analysis). Further conditioning on rs2516448 still left residual association at rs3117027, which is located within the pseudogene *HLA-DPB2* (OR = 1.27, 95% CI = 1.14–1.41, *P* = 7.0 × 10^−6^ compared with OR = 1.28, 95% CI = 1.15–1.42, *P* = 3.1 × 10^−6^ in the unconditional analysis) (Table 1). The LD between these three SNPs is very weak (pairwise *r*^2^ = 0.001), suggesting three independently acting loci. Conditioning on all three SNPs jointly left little evidence for residual association at 6p21.3. The associations with the three loci were further replicated in an independent study including 1140 cervical cancer patients and 1058 controls in the Swedish population 17.

**Table 1:** Association of SNPs and HLA alleles/haplotypes with risk of cervical cancer.

		Allele frequency		Association		
Variants	Alleles^1^	Case	Control	OR	95% CI	*P*
SNPs^2^						
rs9272143	T>C	0.39	0.49	0.65	0.59–0.72	2.8 × 10^−17^
rs2516448 (or rs67841474)	C>T (or →G)	0.59	0.50	1.44	1.30–1.58	5.8 × 10^−13^
rs3117027	C > A	0.35	0.29	1.28	1.15–1.42	3.1 × 10^−6^
HLA alleles/haplotypes^3^						
*HLA-B*						
B^*^0702	–	0.19	0.14	1.42	1.25–1.61	7.9 × 10^−8^
*HLA-DRB1*						
DRB1^*^1301	–	0.04	0.08	0.47	0.37–0.59	3.9 × 10^−10^
DRB1^*^1501	–	0.19	0.15	1.39	1.23–1.57	2.7 × 10^−7^
*HLA-DQA1*						
DQA1^*^0103	–	0.04	0.08	0.49	0.39–0.62	1.9 × 10^−9^
* HLA-DQB1*						
DQB1^*^0603	–	0.04	0.08	0.48	0.38–0.60	5.7 × 10^−10^
DQB1^*^0602	–	0.19	0.15	1.39	1.22–1.58	3.5 × 10^−7^
HLA haplotypes						
DRB1^*^1301-DQA1^*^0103-DQB1^*^0603^4^	–	0.04	0.08	0.47	0.37–0.60	8.8 × 10^−10^
DRB1^*^1501-DQB1^*^0602^5^	–	0.19	0.15	1.39	1.23–1.58	3.8 × 10^−7^

CI, confidence interval; HLA, human leukocyte antigen*;* OR, odds ratio*; P*, two-sided *P* value corresponding to the OR; SNPs, single-nucleotide polymorphisms.

1 Major allele > minor allele for each SNP.

2 Allele frequency corresponds to the minor allele frequency of each SNP, and association results are derived from unconditional logistic regression for each minor allele compared to the wild-type allele with adjustment for the nine informative eigenvectors generated by principal components analysis.

3 Association results are derived from unconditional logistic regression model for each allele/haplotype compared to all the others with adjustment for the nine informative eigenvectors generated by principal components analysis.

4 DRB1^*^1301, DQA1^*^0103, and DQB1^*^0603 are in strong LD with each other and often occur on the same haplotype (pairwise *r*^2^* *≥* *0.97 in the cancer-free controls).

5 DRB1^*^1501 and DQB1^*^0602 are in strong LD and often occur on the same haplotype (pairwise *r*^2^* = *0.98 in the cancer-free controls).

One HLA class I allele (B*0702) and five class II alleles (DRB1*1301, DRB1*1501, DQA1*0103, DQB1*0603, and DQB1*0602) showed evidence of association with cervical cancer at the *P* < 8.1 × 10^−6^ threshold (Table 1). The DRB1*1301-DQA1*0103-DQB1*0603 haplotype conferred reduced risk of cervical cancer (OR = 0.47, 95% CI = 0.37–0.60, *P* = 8.8 × 10^−10^), whereas the B*0702 and DRB1*1501-DQB1*0602 haplotype independently conferred increased risk (OR = 1.42, 95% CI = 1.25–1.61, *P* = 7.9 × 10^−8^; OR = 1.39, 95% CI = 1.23–1.58, *P* = 3.8 × 10^−7^, respectively). When conditioning on B*0702, DRB1*1301-DQA1*0103-DQB1*0603 or DRB1*1501-DQB1*0602, similar strength of association was observed for rs9272143, rs2516448, and rs3117027 as compared to the unconditional analysis, suggesting that the effects of the three SNP loci are independent from the above HLA alleles/haplotypes 17.

SNP rs9272143 was recently reported to be a *cis*-expression quantitative trait locus (eQTL) that affects the expression of *HLA-DRB1* in adipose tissue from 84 Finnish individuals, with the C allele being associated with increased expression of *HLA-DRB1* (*P* = 4.0 × 10^−7^) 22. The expression of *HLA-DRB1* in adipose tissue is probably due to the presence of macrophages, the antigen presenting cells (APCs), in the inflamed adipose tissue. We cannot, however, rule out that the adipocytes themselves may become more proinflammatory with the *HLA-DRB1* expression increasing allele, as the adipocytes are already known to secrete proinflammatory cytokines 23. Consistently, rs9271699, which is in perfect LD (*D′* = 1, *r*^2^ = 1) with rs9272143 in this study, was also found to be a *cis*-eQTL for *HLA-DRB1* in human lymphoblastoid cell line samples from 373 subjects with European ancestry (*P* = 1.1 × 10^−16^) 24, suggesting a role of rs9272143 or its highly correlated variants in regulating *HLA-DRB1* expression. *HLA-DRB1* belongs to the HLA class II beta-chain paralogs, which encodes the *β*-chain of the peptide-antigen receptor HLA-DR and plays a central role in the cell-mediated immune response by presenting processed foreign antigens to CD4+ helper T-lymphocytes 4–6. Impaired HLA class II gene expression has been reported in genital HPV infections and in lesions due to HPV 25,26. It is, hence, biologically plausible that carriers of the C allele of rs9272143, which have higher expression level of *HLA-DRB1,* are less susceptible to cervical cancer. However, further studies are warranted to identify the causal regulatory variation at this locus and evaluate its functional effect in leukocytes and cervical tissues.

Variant rs2516448 is in perfect LD (*D′* = 1, *r*^2^ = 1) with a deletion–insertion polymorphism rs67841474, where the guanine (G) insertion causes a frameshift mutation (known as A5.1) in exon 5 of *MICA*, which in turn results in a truncated MICA protein lacking part of the transmembrane domain (TMD) and the whole cytoplasmic tail. The risk allele T of rs2516448 always occurs together with the frameshift mutation A5.1. *MICA* encodes a membrane-bound protein which acts as a ligand to stimulate an activating receptor, NKG2D, expressed on the surface of essentially all human natural killer (NK), *γδ* T, and CD8^+^ *αβ* T cells 27–29. Normally, MICA is constitutively expressed in low levels on epithelial cells in the gut and thymus, endothelial cells, fibroblasts, and monocytes 30–32, but is upregulated or expressed de novo in stressed conditions, such as during viral and bacterial infections 29,33,34, heat shock 22, DNA damage response 35, oncogenic transformation 27,28, and in autoimmune conditions 36. MICA serves as signal of cellular stress, and engagement of NKG2D by MICA triggers NK cells, and costimulates some *γδ* T cells and antigen-specific CD8^+^ *αβ* T cells, resulting in a range of immune effector functions, such as cytotoxicity and cytokine production 30,37. The recognition of the MICA molecule by the NKG2D receptor enables immune cells to identify and attack infected or transformed cells without the need of MHC class I expression or antigen recognition 38. Thus, the MICA/NKG2D interaction is an effective mechanism for immunosurveillance. The cytoplasmic tail-deleted *MICA-A5.1* gene product is aberrantly transported to the apical surface of human intestinal epithelial cells instead of the basolateral surface, where the interaction with intraepithelial T and NK lymphocytes takes place 39. Furthermore, cervical neoplasia patients carrying the A5.1 allele have less membrane-bound MICA in their lesions 17, which may comprise their ability to alert the immune system of HPV infection or neoplastic change, leading to impaired immune activation and increased risk of tumor development.

The LD estimates between the associated SNPs and classic HLA alleles are shown in Table 2. Although there is a small difference in the LD pattern between the 576 cervical cancer patients with HLA typing data and the 1034 cervical cancer patients with imputed HLA data, the two groups showed comparable correlations between SNPs and HLA alleles. Correlations were observed between rs9272143 and all the cervical cancer-associated HLA alleles, as well as between rs67841474 and B*0702, DRB1*1501 and DQB1*0602 (*r*^2^ > 0). To assess the extent of possible confounding of the association with HLA alleles/haplotypes by the SNP associations, conditional logistic regression analysis was performed for these HLA alleles/haplotypes conditioning on the top SNPs in LD with them. As shown in Table 3, upon conditioning on rs9272143 or rs67841474, associations with both B*0702 and DRB1*1501-DQB1*0602 were significantly attenuated. When conditioning on both rs9272143 and rs67841474, no statistically significant association was observed for B*0702 (conditional analysis: OR = 1.02, 95% CI = 0.88–1.18, *P* = 0.80; unconditional analysis: OR = 1.42, 95% CI = 1.25–1.61, *P* = 7.9 × 10^−8^) or DRB1*1501-DQB1*0602 (conditional analysis: OR = 0.99, 95% CI = 0.86–1.15, *P* = 0.90; unconditional analysis: OR = 1.39, 95% CI = 1.23–1.58, *P* = 3.8 × 10^−7^), suggesting that the associations of these HLA allele/haplotypes are driven by the joint effects of both rs9272143 and rs67841474. The effect of DRB1*1301-DQA1*0103-DQB1*0603 was also attenuated upon conditioning on rs9272143, but there was still statistically significant residual association with decreased risk of cervical cancer (conditional analysis: OR = 0.60, 95% CI = 0.45–074, *P* = 2 × 10^−5^; unconditional analysis: OR = 0.47, 95% CI = 0.37–0.60, *P* = 8.8 × 10^−10^), indicating a direct effect on cervical cancer. The class II HLA molecules encoded by this haplotype may have higher affinity of binding HPV antigens than other HLA alleles.

**Table 2:** Linkage disequilibrium (LD) between associated SNPs and HLA alleles.

	rs9272143		rs67841474		rs3117027	
HLA allele	*D′*	*r*^2^	*D′*	*r*^2^	*D′*	*r*^2^
Controls with imputed HLA data^1^						
B^*^0702	0.50	0.04	0.99	0.16	0.03	0
DRB1^*^1501	1.0	0.17	0.41	0.03	0.01	0
DQB1^*^0602	1.0	0.17	0.43	0.03	0.01	0
DRB1^*^1301	1.0	0.08	0.25	0	0.05	0
DQA1^*^0103	0.99	0.09	0.23	0	0.05	0
DQB1^*^0603	1.0	0.09	0.24	0	0.05	0
Cases with imputed HLA data^1^						
B^*^0702	0.54	0.04	1.0	0.16	0.09	0
DRB1^*^1501	1.0	0.15	0.49	0.04	0.03	0
DQB1^*^0602	1.0	0.15	0.51	0.04	0.03	0
DRB1^*^1301	1.0	0.06	0.15	0	0.03	0
DQA1^*^0103	1.0	0.06	0.07	0	0.05	0
DQB1^*^0603	1.0	0.06	0.12	0	0	0
Cases with direct genotyping data of HLA^2^						
B^*^0702	0.41	0.02	0.96	0.19	0	0
DRB1^*^1501	0.94	0.12	0.61	0.07	0	0
DQB1^*^0602	1.0	0.14	0.61	0.07	0	0
DRB1^*^1301	1.0	0.04	0.28	0	0.48	0
DQB1^*^0603	1.0	0.05	0.27	0	0.42	0

HLA, human leukocyte antigen; SNP, single-nucleotide polymorphism.

1 Classic HLA alleles were imputed from SNP data using HLA^*^IMP 30.

2 Estimated in 278 randomly selected singletons stemming from each family in CervixCan II study by Haploview 21 which have overlapping genotyping data of classic HLA alleles and SNPs.

**Table 3:** Analysis of possible confounding of the HLA allele/haplotypes associations by SNPs.

			B^*^0702			DRB1^*^1501-DQB1^*^0602			DRB1^*^1301-DQA1^*^0103-DQB1^*^0603		
Conditioning on^1^	Case^2^	Control^2^	OR	95% CI	*P*	OR	95% CI	*P*	OR	95% CI	*P*
rs9272143	1034	3948	1.27	1.12–1.45	3 × 10^−4^	1.13	0.99–1.30	0.08	0.60	0.45–0.74	2 × 10^−5^
rs67841474	1034	3946	1.20	1.04–1.38	0.01	1.30	1.12–1.46	2 × 10^−4^	–	–	–
rs9272143 and rs67841474	1034	3946	1.02	0.88–1.18	0.80	0.99	0.86–1.15	0.90	–	–	–

CI, confidence interval; HLA, human leukocyte antigen; SNP, single-nucleotide polymorphism OR, odds ratio; *P*, two-sided *P* value corresponding to the OR.

1 Derived from conditional logistic regression conditioning on specified SNP for each allele/haplotype compared to all the others with adjustment for the nine informative eigenvectors generated by principal components analysis.

2 Number of cervical cancer patients and cancer-free controls, respectively.

These results provide an explanation of both the conflicting findings with regard to variable pattern of association with HLA-B*07 and DRB1*1501-DQB1*0602 and the consistent pattern of association with DRB1*1301-DQA1*0103-DQB1*0603. As shown in Table S1, the LD between the HLA loci (HLA-B*0702 and DRB1*1501) and the SNPs (rs9272143 and rs67841474) varies between ethnically distinct populations. As HLA-B*0702 and DRB1*1501-DQB1*0602 have indirect effects on cervical cancer, variation in the LD pattern between these classic HLA alleles and the causal variants across different populations, could result in different strengths as well as direction of associations. By contrast, DRB1*1301-DQA1*0103-DQB1*0603 has a direct effect and is likely to be pathogenetic. In accordance with the hypothesis that the mechanism of action for causal variants is shared across human populations 40, the biological impact of DRB1*1301-DQA1*0103-DQB1*0603 on the risk for cervical cancer appears to be consistent across populations.

The *HLA-DRB1 cis*-eQTL (rs9272143) and DRB1*1301-DQA1*0103-DQB1*0603 are both located in the MHC class II region and are in complete LD with each other (*D′* = 1), resulting in three haplotypes between these two loci (Table 4). DRB1*1301-DQA1*0103-DQB1*0603 only occurs together with the protective allele C of rs9272143, which has recently been shown to be associated with increased expression of *HLA-DRB1* 22,24. The haplotype *C*-DRB1*1301-DQA1*0103-DQB1*0603 showed the strongest protection against cervical cancer, with carriers having 59% reduced risk as compared with those carrying the most common haplotype *T-others* (OR = 0.41, 95% CI = 0.32–0.52, *P* *=* 6.2 × 10^−13^). This suggests that the class II HLA molecules enclosed by this haplotype have better specificity of binding HPV antigens and are also expressed at a higher level as compared to other alleles, and carriers may therefore benefit from a more efficient presentation of peptides from the causative agent of cervical cancer, HPV.

**Table 4:** Haplotype analysis between rs9272143 and DRB1^*^1301-DQA1^*^0103-DQB1^*^0603.

	Allele frequency		Association^1^		
Haplotype	Case	Control	OR	95% CI	*P*
*T-others*	0.61	0.51	Reference		
*C-others*	0.35	0.41	0.70	0.63–0.78	1.7 × 10^−11^
C-DRB1^*^1301-DQA1^*^0103-DQB1^*^0603	0.04	0.08	0.41	0.32–0.52	6.2 × 10^−13^

CI, confidence interval; OR, odds ratio.

1 Derived from unconditional logistic regression for each haplotype compared to *T-others* with adjustment for the nine informative eigenvectors generated by principal components analysis.

Some limitation in this study should be noted. First, most of the cervical cancer cases included are CIS and only 4.5% are invasive squamous cell carcinoma. We therefore have higher power to detect variants associated with CIS and only limited power to identify associations with invasive carcinoma. Our results apply to CIS, which represent the vast majority of cases, and progression to invasive cancer may require additional, as yet unknown, risk factors. However, CIS and invasive cancer share the same main etiological factor, namely persistent infection by oncogenic types of HPV, indicating that the genetic susceptibility loci identified for CIS will have a similar effect on the two cancer stages. Second, our study did not have information on HPV type. Hence, we are not able to assess if the effect is specific for certain HPV types.

This study was motivated by the variable pattern of association of specific HLA alleles with cervical cancer reported in the literature. Using PCA allowed us to minimize the problem of population stratification. We have found that the associations of cervical cancer risk with B*0702 and DRB1*1501-DQB1*0602 are attributable to the joint effects of a *HLA-DRB1 cis*-eQTL and a frameshift mutation of *MICA*. The mechanism suggested may also explain similar inconsistent results seen between populations for other HLA-associated diseases. On the other hand, the protective haplotype DRB1*1301-DQA1*0103-DQB1*0603 has a direct effect on cervical cancer risk and is expressed at a higher level compared to other classic HLA alleles. Future studies are warranted to identify the specific viral epitopes presented by this haplotype.

## Supporting Information

Table S1 Linkage disequilibrium between HLA alleles and associated SNPs in individuals of CEU, YRI, JPT, and CHB.
