# Supplementary material for: A cis-eQTL of HLA-DRB1 and a frameshift mutation of MICA contribute to the pattern of association of HLA alleles with cervical cancer
Source: Cancer Med. 2014 Feb 12;3(2):445–52. doi: 10.1002/cam4.192 (PMC3987094; doi:10.1002/cam4.192)
Supplement: Table S1 — Linkage disequilibrium between HLA alleles and associated SNPs in individuals of CEU, YRI, JPT, and CHB. [file cam40003-0445-sd1.docx]

**Supplementary Table S1. Linkage disequilibrium between HLA alleles and associated SNPs in individuals of CEU, YRI, JPT and CHB.**

|  |  |  | **rs9272143** ^c^ | |  | **rs67841474** ^d^ | |
| --- | --- | --- | --- | --- | --- | --- | --- |
| **HLA allele** | **Allele frequency** | **Tag SNP/haplotype** ^b^ | ***D’*** | ***r^2^*** |  | ***D’*** | ***r^2^*** |
| ***B*0702*** ^a^ |  |  |  |  |  |  |  |
| CEU | 0.183 | - | 0.42 | 0.02 |  | 1.0 | 0.14 |
| YRI | 0.075 | - | 0 | 0 |  | 0.63 | 0.17 |
| JPT | 0.057 | - | 1.0 | 0.12 |  | 1.0 | 0.34 |
| CHB | 0 | - | NA | NA |  | NA | NA |
| ***DRB1*1501*** ^b^ |  |  |  |  |  |  |  |
| CEU | 0.200 | rs3135388A (*D’*=0.97,*r^2^*=0.99) | 1.0 | 0.14 |  | 0.76 | 0.07 |
| YRI | 0.008 | rs443623A (*D’*=1,*r^2^*=1) | 1.0 | 0.01 |  | NA | NA |
| JPT | 0.102 | rs7773756T_rs6919855G_rs6901830G(*D’*=1,*r^2^*=1) | 1.0 | 0.05 |  | 1.0 | 0.02 |
| CHB | 0.067 | rs7773756T_rs6903608G_rs620202C (*D’*=1,*r^2^*=1) | 1.0 | 0.07 |  | 0.06 | 0 |

CEU, Utah residents with Northern and Western European ancestry; YRI , Yoruba in Ibadan, Nigeria; JPT, Japanese in Toyko, Japan; CHB, Han Chinese in Beijing, China.

NA, not available.

^a^ Classic *HLA-B* data was generated by next-generation sequencing through exon 2 and 3 of *HLA-B* in Hapmap subjects of CEU, YRI, JPT and CHB in a previous study (41).

Pairwise LD between *B*0702* and two SNPs (rs9272143 and rs67841474) was examined by Haploview (21) in subjects with overlapping data of SNPs and classic *HLA-B*

alleles.

^b^ Classic *HLA-DRB1* data was not public available. Tag SNPs/haplotypes for *HLA-DRB1*1501* were identified in Hapmap subjects of CEU, YRI, JPT and CHB, respectively in a

previous study (15). Genotyping data of the tag SNPs/haplotypes was obtained from Phase III Hapmap data (42) and the 1000 Genome Project (43). Pairwise LD between tag

SNPs/haplotypes for *HLA-DRB1*1501* and two SNPs (rs9272143 and rs67841474) was examined by Haploview (21) in subjects with overlapping genotyping data. rs443623

is monomorphic in YRI of the 1000 Genome Project (43) hence its LD with rs67841474 in YRI was not able to be examined.

^c^ Genotyping data of rs9272143 in CEU, YRI, JPT and CHB was obtained from Phase III Hapmap data (42).

^d^ Genotyping data of rs67841474 in CEU, YRI, JPT and CHB was obtained from the 1000 Genome Project (43).

**References**

41. Erlich RL, Jia X, Anderson S, Banks E, Gao X, Carrington M, Gupta N, DePristo MA, Henn MR, Lennon NJ, de Bakker PI. Next-

generation sequencing for HLA typing of class I loci. *BMC Genomics* 2011;**12**:42.

42. International HapMap 3 Consortium, Altshuler DM, Gibbs RA, Dermitzakis E, Schaffner SF, Yu F, Peltonen L, Dermitzakis E, Bonnen PE,

Altshuler DM, Gibbs RA, de Bakker PI, et al. Integrating common and rare genetic variation in diverse human populations. *Nature* 2010;

**467**:52-58.

43. 1000 Genomes Project Consortium. A map of human genome variation from population-scale sequencing. *Nature*  2010; **467**: 1061-73.
